# Effect on Satisfactory Seizure Control and Heart Rate Variability of Thread-Embedding Acupuncture for Drug-Resistant Epilepsy: A Patient-Assessor Blinded, Randomized Controlled Trial

**DOI:** 10.1155/2023/5871991

**Published:** 2023-09-19

**Authors:** Van-Dan Nguyen, Duc-Thang Pham, Minh-An Thuy Le, Guo-Ming Shen

**Affiliations:** ^1^School of Integrated Chinese and Western Medicine, Anhui University of Chinese Medicine, Hefei, 230012 Anhui Province, China; ^2^Faculty of Traditional Medicine, University of Medicine and Pharmacy at Ho Chi Minh City, Ho Chi Minh City 100000, Vietnam; ^3^Department of Neurology, University of Medicine and Pharmacy at Ho Chi Minh City, Ho Chi Minh City 100000, Vietnam; ^4^Institute of Integrated Chinese and Western Medicine, Anhui University of Chinese Medicine, Hefei, 230012 Anhui Province, China

## Abstract

This randomized controlled trial investigates the efficacy of thread-embedding acupuncture (TEA) compared to sham TEA in treating drug-resistant epilepsy (DRE). Fifty-four DRE outpatients were randomly divided into two groups: TEA (27 patients) and sham TEA (27 patients). Both groups received four sessions of TEA or sham TEA, spaced four weeks apart, targeting GV20, GV14, BL15, BL18, ST40, and GB34 acupoints. Antiseizure medications were maintained at consistent doses throughout the study. Outcome measures included satisfactory seizure control, seizure freedom, and heart rate (HR) and heart rate variability (HRV) measurements. TEA demonstrated a significantly higher rate of satisfactory seizure control at follow-up compared to the sham TEA group (37% vs. 3.7%, *p* = 0.003). While no significant intergroup differences were observed in HR, HRV, and HRV components at each stage, the TEA group experienced a significant decrease in HR and a significant increase in HRV posttreatment. This study demonstrates TEA's effectiveness in managing DRE and suggests its impact may relate to heightened parasympathetic nerve activity. Further research with extended follow-up periods is necessary to validate these findings.

## 1. Introduction

Epilepsy, a chronic neurological disorder affecting individuals of all ages and genders across the globe [1], is characterized by a heightened susceptibility to epileptic seizures. Most seizures can be effectively managed with antiseizure medications (ASMs) [1]. Nonetheless, a significant portion of the epilepsy population, approximately one-third, continues to grapple with seizures despite ASM treatment [2, 3]. Individuals who do not attain seizure freedom following appropriate trials of two well-tolerated and appropriately chosen ASM regimens, whether administered as monotherapy or in combination, are categorized as having drug-resistant epilepsy (DRE) [2]. In Vietnam, epilepsy's prevalence spans from 4.4 to 14 per 1000 individuals [4, 5], with approximately 74% of patients residing in rural areas [6]. A prevailing issue among individuals with DRE is the impairment of parasympathetic activity, which has been linked to the occurrence of sudden unexpected death in epilepsy [7, 8].

Vagus nerve stimulation (VNS) is an invasive technique sanctioned by the Food and Drug Administration to manage DRE. It entails the stimulation of the vagus nerve, a pivotal element of the parasympathetic nervous system [9]. Numerous studies have scrutinized alterations in autonomic nerve function before and after VNS surgery [10]. However, VNS remains untapped for DRE treatment in Vietnam due to resource constraints, including specialized personnel and facilities, compounded by its high cost [6]. Consequently, the prevailing approach to managing DRE in Vietnam primarily involves ASM combinations. The imperative lies in uncovering more efficacious and advantageous strategies for DRE management.

In Vietnam, acupuncture using different methods is widely practiced, covered by Vietnam Health Insurance, and endorsed by the Vietnam Ministry of Health for treating various health issues. One of these methods is TEA, a type of acupuncture that involves inserting a needle attached to a dissolvable thread into specific points on the body. While TEA works similarly to traditional acupuncture, it has certain benefits like stronger stimulation and a longer-lasting treatment effect [11]. The effectiveness of TEA is attributed to the gradual breakdown of the thread. The Ministry of Health has approved TEA for managing various conditions, including epilepsy. In our previous study, which had a smaller group of 30 patients with drug-resistant epilepsy (DRE) [12], we found that TEA was safe with no serious side effects. We observed that the TEA group had fewer seizures and improved scores on the Quality of Life in Epilepsy Inventory (QOLIE-31) and the National Hospital Seizure Severity Scale (NHS3) compared to the group that received a sham TEA. A previous study has also shown that TEA can boost parasympathetic nerve activity [13]. Yet, no research has explored how TEA affects the autonomic nervous system in DRE patients. This led us to conduct a randomized controlled trial to assess the impact of TEA on treating DRE, comparing it to a sham TEA procedure. We also investigated changes in heart rate variability (HRV), which reflects the function of the autonomic nervous system, before and after TEA.

## 2. Materials and Methods

### 2.1. Study Design

This study was structured as a randomized controlled trial, with both patients and outcome assessors blinded. It adopted a two-arm parallel design, with eligible participants randomly assigned to either the TEA group or the sham TEA group in a 1 : 1 ratio. The study protocol gained approval from the Ethics Committee of Nguyen Tri Phuong Hospital in Ho Chi Minh City, Vietnam (approval no. 1284/NTP-CĐT). All procedures adhered to the ethical guidelines set forth in the Declaration of Helsinki for medical research involving human subjects. Our study methodology drew on previous work by V.-D.N. outlined in a prior protocol (ClinicalTrials.gov, NCT04673071). The reporting of the study followed the guidelines specified in the Revised Standards for Reporting Interventions in Clinical Trials of Acupuncture (STRICTA), while also extending compliance with the CONSORT statement [14].

### 2.2. Study Population

Outpatients were enrolled between March 2022 and December 2022 in the Epilepsy Clinic within the Department of Neurology. All included participants fulfilled the International League Against Epilepsy (ILAE) defined criteria for DRE. Eligibility criteria encompassed both male and female DRE patients aged 18 to 60, who had experienced at least one seizure within the preceding three months.

Exclusion criteria encompassed individuals who had undergone previous epilepsy surgery, displayed skin disorders (characterized by swelling, warmth, and redness) at acupoints, or had hemostasis disorders (with an international normalized ratio > 2.0 or ongoing use of anticoagulants). Pregnant individuals were also excluded, along with those suffering from conditions like extreme fatigue, severe gastrointestinal disorders, cardiovascular complications, diabetes mellitus, kidney disorders, liver diseases, thyroid malfunctions, acute stroke, or brain tumors. Individuals who had undergone TEA within the past six months were not included, as well as those diagnosed with conditions like schizophrenia, depression, anxiety, or alcoholism.

### 2.3. Randomization and Blinding

A simple randomization approach was employed for participant allocation. This entailed using envelopes containing both even- and odd-numbered sequences, where odd numbers corresponded to the intervention group and even numbers to the control group. The random number generation was performed using Microsoft Excel®. To maintain confidentiality, a designated study member (D.-T.P.) retained the generated random table in a secured file with restricted access.

The procedural aspects of both interventions were indistinguishable, differing solely in the presence or absence of a polydioxanone (PDO) thread within the needles. The intervention group underwent TEA using needles equipped with PDO threads, while the control group received sham TEA with needles lacking PDO threads. Following needle removal, no observable dissimilarities were evident between the two interventions. Both the patients and the outcome assessor remained uninformed regarding the treatment received by the participants.

### 2.4. Intervention

The TEA and sham TEA interventions were conducted by a seasoned traditional medicine physician (V.-D.N.) with a decade of acupuncture experience. The procedures took place at specific acupoints, including Baihui (GV20), Dazhui (GV14), bilateral Xinshu (BL15), Ganshu (BL18), Fenglong (ST40), and Yanglingquan (GB34). The techniques for TEA and sham TEA were parallel, differing only in the presence or absence of a PDO thread within the needles. The choice of these acupoints followed the guidelines provided by the Vietnamese Ministry of Health, alongside previous research exploring the use of TEA for epilepsy patients [7, 15, 16]. In this study, monoshaped TEA with a 31G-30 mm needle and a PDO thread (7-0 USP size, 30 mm) (JBP V line; Feel-Tech Co., Ltd., JBP Korea, Republic of Korea) were employed.

All interventions were conducted in a room maintained at 26 ± 1°C between 01:30 PM and 04:30 PM, with the patient positioned in the prone posture. Adhering to the clean needle technique, the intervention sites were disinfected using 70% alcohol. Needles were inserted perpendicular at BL15, BL18, GB34, and GV14, while an oblique angle toward the nose was employed at GV20, all to a depth of 3 cm. Notably, no stimulation techniques were applied to elicit the “De Qi” (needling sensation).

Each participant underwent a total of four TEA or sham TEA sessions, spaced four weeks apart. Throughout the study, ASMs were maintained at the prescribed fixed dosage by neurologists for both groups.

### 2.5. Follow-Up

**Figure 1:**
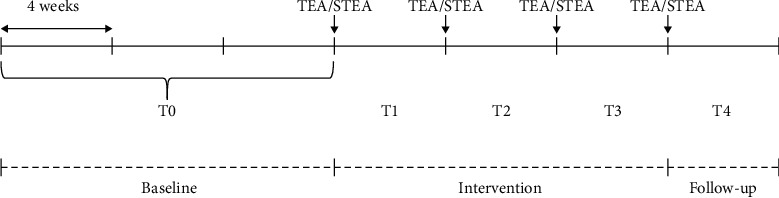
Study protocol. T0: baseline; T1-T3: intervention (TEA/sham TEA); T4: follow-up; TEA: thread-embedding acupuncture; STEA: sham thread-embedding acupuncture.

The study encompassed three distinct periods: a 12-week baseline phase (T0), a 12-week intervention phase (T1–T3), and a 4-week follow-up phase (T4) (Figure 1). Each participant underwent a total of six visits at the study site, which comprised an initial baseline visit, four intervention visits, and a follow-up visit. During each of these encounters, a neurologist (M.-A.T.L.) evaluated the patient's seizure status for the preceding four weeks, noting the frequency of epilepsy seizures. Additionally, heart rate (HR) and heart rate variability (HRV) were measured.

After each procedure, patients were observed at the clinic for a duration of 30 minutes. Subsequently, if no adverse events were observed, they were cleared for discharge. Patients, along with their caregivers, were educated on the importance of self-monitoring seizure conditions and documenting any adverse events at home. Adverse events commonly associated with TEA included swelling, ecchymosis, tenderness, pain, infection, dimple formation, thread extrusion, and foreign body reaction [17, 18].

### 2.6. Outcomes

The study included three efficacy outcomes: satisfactory seizure control, seizure freedom, and the measurement of HR and HRV.

Satisfactory seizure control was ascertained when the count of epilepsy seizures within the past four weeks during each intervention visit (T1-T4) exhibited a decline of 50% or more in comparison to the seizure count over the four weeks at the baseline (T0) stage.

Patients were considered to have achieved seizure freedom if they did not encounter any seizures by the follow-up visit (T4) of the study.

HR and HRV assessments were conducted as previously described [19]. Participants rested for ten minutes before HR and HRV monitoring commenced. A photoplethysmography device (Kyto Electronic Co., China) affixed to the right earlobe was employed to measure HR and HRV, with readings taken every five minutes. HRV pertains to the variation in time intervals between consecutive heartbeats or successive R-waves of the QRS signal on the electrocardiogram, measured in milliseconds. In addition, we recorded the “time” domain variables such as the standard deviation of RR interval (SDNN) and the square root of mean squares of differences between RR intervals (RMSSD). The “frequency” domain was also recorded including low-frequency (LF) values spanning from 0.04 to 0.15 Hz and high-frequency (HF) values ranging from 0.15 to 0.4 Hz.

### 2.7. Sample Size Calculation

Sample size determination followed a comparison of two-proportion approach. The estimated proportions of seizure freedom in the TEA and sham TEA groups were 46% and 12%, respectively, as indicated by Chen et al.'s study [20]. With a power of 80% and a type I error rate of 5%, the requisite sample size equated to 25 patients per group. Considering an estimated 10% attrition, the total sample size was set at 54.

### 2.8. Statistical Analysis

Data analysis was conducted using Stata software version 15.0 (StataCorp, College Station, TX). Quantitative variables were presented as median along with the interquartile range due to skewed distributions. Qualitative variables were represented using frequencies and percentages. For comparing baseline patient characteristics and outcomes, the Mann–Whitney *U*-test was employed for quantitative variables and Fisher's exact test for qualitative variables. HR, HRV, and its constituents (SDNN, RMSSD, LF, and HF) were contrasted before and after treatment within each group through the utilization of the Wilcoxon signed-rank test. A significance threshold of *p* values less than 0.05 was adopted.

## 3. Results

### 3.1. Baseline Characteristics

**Figure 2:**
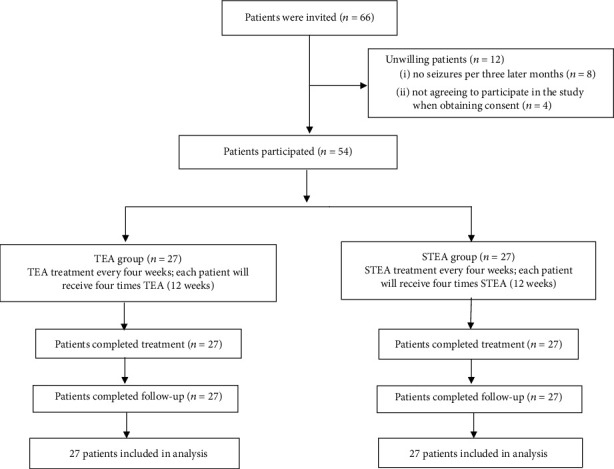
Flowchart of the participants through the study. TEA: thread-embedding acupuncture; STEA: sham thread-embedding acupuncture

Initially, 66 participants were evaluated for eligibility. Among them, eight individuals had experienced no seizures in the past three months, and an additional four declined participation after consent was given. Ultimately, 54 eligible patients underwent randomization, and none withdrew from the study (Figure 2).

Both study groups exhibited balanced baseline characteristics (Table 1). Female participants constituted the majority in both the TEA (59.26%) and sham TEA (55.56%) groups. The median age at enrollment was 31 for the TEA group and 32 for the sham TEA group, with age ranges spanning from 19 to 58 years. The median age at the onset of the first seizure was 18 and 20 years, respectively. The median number of seizures over four weeks at baseline was 4 for both groups.

### 3.2. Satisfactory Seizure Control

Following four treatment sessions, a notable distinction in satisfactory seizure control emerged between the two groups (Table 2). Within the TEA group, ten participants (37.04%) achieved satisfactory seizure control. Among these, seven individuals encountered focal seizures, while the rest experienced focal seizures transitioning to bilateral tonic-clonic seizures. Conversely, within the sham TEA group, a solitary participant (3.70%) with focal seizures achieved satisfactory seizure control. Nonetheless, no statistically significant variance was observed regarding seizure freedom, even though the TEA group exhibited a higher rate of seizure freedom (2 patients, 7.41%) compared to the sham TEA group (0%).

### 3.3. Heart Rate and Heart Rate Variability

HR and HRV values exhibited no significant differences between the two groups at each stage (Table 3). However, a noteworthy pattern emerged: the TEA group demonstrated a significant decrease in HR and a significant increase in HRV from baseline to T3 and T4. In contrast, the changes in HR and HRV were more subtle in the sham TEA group and were not statistically significant.

The fluctuations in the “time” and “frequency” domains are outlined in Table 4. No significant disparities were observed between the two groups in any of the HRV components (SDNN, RMSSD, LF, and HF) at each stage. Furthermore, minimal alterations in any of the HRV components were noted from baseline to each stage, apart from RMSSD at the end of the follow-up in the TEA group.

### 3.4. Safety Assessment

Mild pain at the intervention sites was reported by all participants in both groups, but it subsided within 15 minutes. Among these, four participants in the TEA group and three in the sham TEA group also reported mild ecchymosis, which resolved within five days without requiring intervention. No other adverse events were documented in either group.

## 4. Discussion

Our study underscores a notable improvement in the rate of satisfactory seizure control within the TEA group, as compared to the sham TEA group. Furthermore, the TEA group exhibited significant decreases in HR and significant increases in HRV from baseline to postintervention. It is worth noting that no substantial differences emerged between the two groups concerning HR, HRV, and their respective components.

The selection of acupoints for the treatment of epilepsy has displayed variation across different studies [15, 16, 21–26]. Within the context of this trial, a combination of commonly employed acupoints for epilepsy treatment, namely, Dazhui (GV14), Xinshu (BL15), and Fenglong (ST40) [26], was integrated with Baihui (GV20), Ganshu (BL18), and Yanglingquan (GB34). Baihui serves to clear the mind, uplift spirits, enhance yang energy, alleviate inner turbulence, and foster revival. Ganshu, associated with the liver, aims to disperse wind, pacify the liver, facilitate Qi movement, and mitigate distress. Yanglingquan's function is to disperse damp-heat, eliminate channel obstructions, relax tendons, and regulate rebellious Qi. This amalgamation of acupoints can potentially synergize the reduction of tangible or intangible phlegm, the cessation of inner turbulence, the harmonization of Qi circulation, the activation of channels, and the correction of common Zang-fu dysfunctions. These effects hold promise in addressing the underlying causes of epilepsy, referred to as “Dian Xian.” This disorder encompasses phlegm accumulation, emotional imbalances, and Qi irregularities, which can lead to Zang-fu imbalances, disruption of Yin and Yang equilibrium, and blockages in Qi and blood flow along meridians, within the brain, and across Zang-fu organs [22, 25, 27].

The pathogenesis of DRE is intricate, and its treatment presents a current challenge [28, 29]. Employing ASMs alone or in conjunction with other Western medical approaches often falls short of fully controlling seizures. The thalamus, a crucial cerebral structure in both the onset and spread of DRE, also plays a vital role in the mechanism of acupuncture [25]. Investigations involving various acupuncture points have highlighted their effects on the thalamus. For instance, stimulating the Dazhui (GV14) point has been shown to mainly inhibit epileptiform activities prompted by pentylenetetrazole (PTZ) within ventrobasal thalamic neurons [30]. Similarly, stimulating the Yanglingquan (GB34) point can modulate thalamic gene expression [31]. TEA shares a mechanism of action with acupuncture, but its effects are prolonged due to the gradual dissolution of the medical thread, leading to sustained acupoint stimulation [11, 21]. Earlier studies have explored using TEA with catgut thread for treating epilepsy and DRE [15, 16, 21–24]. These mechanisms encompass alterations in nerve potential and membrane ion channels, diminution of neural circuits during seizures, induction of immune-inflammatory responses, release of active substances like serotonin and arachidonic acid, and reduction of apoptotic neurons in the hippocampus through inhibition of P53 protein and elevation of bcl-2 protein expression. Furthermore, polydioxanone (PDO), a synthetic monofilament polymer derived from polydioxanone polymer, is a safe and well-tolerated bioavailable material. It notably exerts no significant adverse effects on immune function and exhibits a propensity to evoke an anti-inflammatory response upon prolonged in vivo exposure [32]. Our study findings affirm the safety of TEA intervention, with mild and transient adverse events (AEs). These outcomes align with previous reports on TEA's safety profile [12, 17, 18].

Our study also observed a reduction in parasympathetic system activity, consistent with earlier investigations [8, 33, 34]. The value of HRV offers insights into the equilibrium within the autonomic nervous system (ANS) [35]. Elevated HRV values signify a prevalence of parasympathetic activity, while decreased HRV values suggest sympathetic predominance, influenced by both internal and external stimuli modulating the ANS. Autonomic dysregulation is a prevalent issue among DRE patients [8], with a tendency toward parasympathetic system hypoactivity. In our research, HR decreased and HRV increased after the interventions, although most HRV components exhibited no significant alterations, except for RMSSD. RMSSD is identified as a biomarker for epilepsy severity, with low values linked to a heightened risk of sudden unexpected death [33, 36]. The increase in RMSSD post-TEA suggests a potential rise in parasympathetic nerve activity, aligning with earlier findings [13]. Notably, manual acupuncture at acupoints like Xinshu (BL15) [37] or Baihui (GV20) [38] prompts parasympathetic nervous system activation, resulting in lowered heart rate and increased HRV. Moreover, diverse acupoint stimulation through various acupuncture techniques has demonstrated the ability to enhance parasympathetic tone and regulate thalamic neuronal activity [39]. TEA's efficacy in treating seizures may be partially attributed to the bolstered parasympathetic tone.

The study presents several limitations. Firstly, the seizure diaries might not have accounted for seizures occurring during sleep, indicating a potential underreporting. Future research should consider incorporating video monitoring during sleep to address this issue. Secondly, the photoplethysmography device's capacity to capture heart rate variations throughout the day is constrained, potentially missing the full impact of diurnal rhythm on HRV. To enhance accuracy, standardized methodologies for HRV measurement and reporting in epilepsy patients are warranted. Thirdly, the study exclusively included participants with focal seizures, leaving out other seizure types like absence seizures or tonic/clonic seizures. Lastly, due to resource and time constraints, the study period was relatively short. To comprehensively assess TEA's effectiveness for epilepsy, future investigations should encompass a broader spectrum of epilepsy types and incorporate an extended follow-up period.

## 5. Conclusions

TEA demonstrates enhanced satisfactory seizure control in individuals with DRE as compared to sham TEA. Furthermore, there was a significant decrease in HR and increase in HRV within the TEA group. To establish a more comprehensive understanding of TEA's clinical efficacy when combined with ASMs for DRE management, further rigorous randomized controlled trials are needed. These trials should incorporate acupoint combinations grounded in traditional medicine syndromes, extend the intervention and follow-up periods, and adhere to methodological rigor.

## Figures and Tables

**Table 1:** Characteristics at baseline of subjects treated with TEA or sham TEA.

	TEA (*n* = 27)	STEA (*n* = 27)	*p* value^a^
Gender (*n*, %)			
Male	11 (40.74)	12 (44.44)	0.783
Female	16 (59.26)	15 (55.56)	
Education (*n*, %)			
Secondary school	12 (44.44)	8 (29.63)	0.484
High school	9 (33.33)	10 (37.04)	
University degree	6 (22.22)	9 (33.33)	
Employment (*n*, %)			
Unemployed	9 (33.33)	9 (33.33)	1.000
Intellectual labor	7 (25.93)	7 (25.93)	
Manual labor	11 (40.74)	11 (40.74)	
Age (years, median (IQR))	31 (27–39)	32 (25–38)	0.795
BMI (kg/m^2^, mean ± SD)	20.96 ± 1.43	20.49 ± 1.58	0.260
Age onset (years, median (IQR))	18 (10–30)	20 (12–28)	0.467
Age diagnosis (years, median (IQR))	18 (12–30)	20 (16–28)	0.359
Seizure type^b^ (*n*, %)			
Focal seizures	17 (62.96)	19 (70.37)	0.564
Focal seizures to bilateral tonic-clonic	14 (51.85)	15 (55.56)	0.784
Number of seizures (median (IQR))	4 (3–5)	4 (3–5)	0.986
Number of antiepileptic drugs in use (median (IQR))	2 (2–3)	2 (2–3)	0.829
SBP (mmHg, median (IQR))	116 (103–121)	111 (108–120)	0.696
DBP (mmHg, median (IQR))	73 (69–76)	72 (68–79)	0.945

Note: ^a^Mann–Whitney *U*-test for comparison of medians, *χ*^2^ test for comparison of proportions. ^b^Seizure type was based on ILAE 2017. TEA: thread-embedding acupuncture; STEA: sham thread-embedding acupuncture; SBP: systolic blood pressure; DBP: diastolic blood pressure; BMI: body mass index; IQR: interquartile range; SD: standard deviation.

**Table 2:** Satisfactory seizure control rate.

Stage	Satisfactory seizure control^∗^ (no. of participants (%))			Seizure freedom (no. of participants (%))		
	TEA (*n* = 27)	STEA (*n* = 27)	*p* value^a^	TEA (*n* = 27)	STEA (*n* = 27)	*p* value^a^
T1-T0	0	0	—	0	0	—
T2-T0	1 (3.70%)	0	0.5	0	0	—
T3-T0	4 (14.81%)	0	0.055	1 (3.70%)	0	0.5
T4-T0	10 (37.04%)	1 (3.70%)	0.003	2 (7.41%)	0	0.245

Note: TEA: thread-embedding acupuncture; STEA: sham thread-embedding acupuncture; T0: baseline; T1-T3: intervention; T4: follow-up. ^a^Fisher's exact test for comparison of rate between the TEA and sham TEA groups. ^∗^Satisfactory seizure control including seizure freedom.

**Table 3:** Heart rate and heart rate variability.

Stage	HR (bpm) (median (IQR))			*p* value^b^		HRV (ms) (median (IQR))			*p* value^b^	
	TEA	STEA	*p* value^a^	TEA	STEA	TEA	STEA	*p* value^a^	TEA	STEA
T0	70 (65–78)	71 (67–76)	0.972			36 (28–45)	35 (28–46)	0.802		
T1	70 (66–76)	71 (68–76)	0.697	0.772	0.517	38 (28–45)	35 (28–46)	0.634	0.491	0.666
T2	69 (63–78)	71 (66–75)	0.911	0.942	0.159	39 (30–45)	36 (29–45)	0.467	0.075	0.090
T3	67 (62–75)	69 (66–73)	0.238	0.005	0.061	41 (31–45)	37 (30–45)	0.573	0.016	0.070
T4	65 (63–76)	71 (68–75)	0.203	0.018	0.324	42 (32–45)	38 (32–45)	0.521	0.008	0.053

Note: HR: heart rate; HRV: heart rate variability; IQR: interquartile range; TEA: thread-embedding acupuncture; STEA: sham thread-embedding acupuncture; T0: baseline; T1-T3: intervention; T4: follow-up. ^a^Mann–Whitney *U*-test for comparison of medians between the TEA and sham TEA groups. ^b^Wilcoxon signed rank-sum test for comparison of median changes from baseline to each stage (T1–T4) in the TEA group and STEA group.

**Table 4:** The variation of the time and frequency domain of heart rate variability.

Stage	SDNN (median (IQR))			*p* value^b^		RMSSD (median (IQR))			*p* value^b^		LF (median (IQR))			*p* value^b^		HF (median (IQR))			*p* value^b^	
	TEA	STEA	*p* value^a^	TEA	STEA	TEA	STEA	*p* value^a^	TEA	STEA	TEA	STEA	*p* value^a^	TEA	STEA	TEA	STEA	*p* value^a^	TEA	STEA
T0	47.04 (41.32–67.65)	48.77 (36.40–65.34)	0.911			28.80 (22.17–32.66)	26.74 (19.75–38.38)	0.802			286.16 (234.14–438.04)	237.42 (135.49–561.92)	0.528			185.95 (116.42–357.08)	240.02 (145.06–364.64)	0.494		
T1	55.41 (36.62–65.49)	53.02 (40.12–62.94)	0.993	0.773	0.532	27.98 (17.66–36.25)	25.33 (20.55–39.80)	0.993	0.719	0.962	333.83 (190.06–477.91)	264.58 (185.21–486.84)	0.870	0.885	0.719	241.21 (100.69–404.33)	272.03 (189.82–356.65)	0.484	0.755	0.866
T2	53.27 (32.89–74.31)	55.95 (38.2–64.17)	0.815	0.923	0.464	28.25 (17.99–37.70)	27.36 (21.39–37.26)	0.904	0.374	0.105	301.54 (214.58–609.25)	382.79 (217.72–485.23)	0.473	0.532	0.325	195.37 (138.69–385.37)	234.05 (165.21–376.97)	0.611	0.471	0.923
T3	48.95 (42.85–75.91)	53.42 (44.45–62.27)	0.742	0.118	0.486	28.27 (21.31–39.70)	27.98 (18.95–41.50)	0.938	0.230	0.471	342.68 (211.61–523.60)	409.73 (245.23–528.38)	0.647	0.773	0.414	252.31 (156.28–367.29)	250.67 (176.37–340.44)	0.924	0.259	0.361
T4	55.95 (42.59–74.46)	53.07 (46.35–67.77)	0.539	0.203	0.203	33.73 (24.6–46.14)	27.15 (23.54–45.75)	0.392	*0.008*	0.195	354.52 (283.66–506.74)	411.83 (289.04–545.48)	0.775	0.068	0.164	260.32 (201.21–357.26)	238.93 (211.00–394.77)	0.802	0.259	0.124

Note: SDNN: standard deviation of RR interval; RMSSD: root square root of mean squares of differences between RR intervals; LF: low frequency; HF: high frequency; IQR: interquartile range; TEA: thread-embedding acupuncture; STEA: sham thread-embedding acupuncture; T0: baseline; T1-T3: the intervention; T4: follow-up. ^a^Mann–Whitney *U*-test for comparisons of medians between the TEA and sham TEA groups. ^b^Wilcoxon signed rank-sum test for comparisons of median changes from baseline to each stage (T1–T4) in the TEA group and sham TEA group.

## Data Availability

The data that support the findings of this study are available from the corresponding author upon reasonable request.

## Supplementary Materials

Supplementary Materials Table S1: details of acupuncture treatment based on the STRICTA 2010 checklist. Table S2: the CONSORT 2010 checklist with the Nonpharmacological Trials Extension to CONSORT.
