# Supplementary material for: Effect on Satisfactory Seizure Control and Heart Rate Variability of Thread-Embedding Acupuncture for Drug-Resistant Epilepsy: A Patient-Assessor Blinded, Randomized Controlled Trial
Source: Behav Neurol. 2023 Sep 19;2023:5871991. doi: 10.1155/2023/5871991 (PMC10522444; doi:10.1155/2023/5871991)
Supplement: Supplementary Materials — Table S1: details of acupuncture treatment based on the STRICTA 2010 checklist. Table S2: the CONSORT 2010 checklist with the Nonpharmacological Trials Extension to CONSORT. [file 5871991.f1.zip › update_Table S1. the STRICTA 2010 checklist.docx]

**Table S1. Details of acupuncture treatment based on the STRICTA 2010 checklist**

| **Item** | **Detail** | **Reported on Line No** |
| --- | --- | --- |
| **1. Acupuncture rationale** | 1a) Style of acupuncture (e.g. Traditional Chinese Medicine, Japanese, Korean, Western medical, Five Element, ear acupuncture, etc) | Thread embedding acupuncture (TEA) |
|  | 1b) Reasoning for treatment provided, based on historical context, literature sources, and/or consensus methods, with references where appropriate | Have reasoning for treatment provided: based on Vietnamese Ministry of Health guideline, historical context, previous studies on TEA for patients with epilepsy and drug-resistant epilepsy |
|  | 1c) Extent to which treatment was varied | No. |
| **2. Details of needling** | 2a) Number of needle insertions per subject per session (mean and range where relevant) | 10 needle |
|  | 2b) Names (or location if no standard name) of points used (uni/bilateral) | The six TEA points used in this study are GV20, BL15, BL18, ST40, GV14 and GB34   - Baihui  (GV20): The midpoint of the connecting line between the auricular apices - Xin Shu (BL15): In the upper back region, at the same level as the inferior border of the spinous process of the fifth thoracic vertebra (T5), 1.5 B-cun lateral to the posterior median line - Gan Shu (BL18): In the upper back region, at the same level as the inferior border of the spinous process of the ninth thoracic vertebra (T9), 1.5 B-cun lateral to the posterior median line. - Fenglong (ST40): On the anterolateral aspect of the leg, lateral border of the tibialis anterior muscle, 8 B-cun superior to the prominence of the lateral malleolus - Dazhui (GV14): In the posterior region of the neck, in the depression inferior to the spinous process of the seventh cervical vertebra (C7), on the posterior median line - Yanglingquan (GB34): On the fibular aspect of the leg, in the depression anterior and distal to the head of the fibula   The description of acupoints was made according to international standard terminology of WHO (2008).  WHO Regional Office for the Western Pacific. *WHO Standard Acupuncture Point Locations in the Western Pacific Region*; World Health Organization: Manila, Philippines, 2008 |
|  | 2c) Depth of insertion, based on a specified unit of measurement, or on a particular tissue level | A depth of 3 cm and angles of the needle depending on acupoints (perpendicular at BL15, BL18, GB34 and GV14; and oblique toward nose at GV20) |
|  | 2d) Response sought (e.g. *de qi* or muscle twitch response) | The Traditional medicine doctor will insert 10 disposable sterile TEA needle, and immediately withdraws it, without further stimulation and manual manipulation for Deqi  will not be allowed |
|  | 2e) Needle stimulation (e.g. manual, electrical) |  |
|  | 2f) Needle retention time |  |
|  | 2g) Needle type (diameter, length, and manufacturer or material) | Mono-shaped TEA with needle (31G-30 mm) and polydioxanone thread (7-0 USP size, 30 mm) (JBP V line; Feel-tech Co*.,*Ltd*,*JBP Korea, Republic of Korea) |
| **3. Treatment regimen** | 3a) Number of treatment sessions | The study included a baseline period of 16 weeks, a treatment period of 12 weeks and follow-up the outcome data after another 4 weeks |
|  | 3b) Frequency and duration of treatment sessions |  |
| **4. Other components of treatment** | 4a) Details of other interventions administered to the acupuncture group (e.g. moxibustion, cupping, herbs, exercises, lifestyle advice) | Anti-epileptic drugs were maintained at the fixed dose prescribed by the Western neurologists for both study groups for the duration of the study |
|  | 4b) Setting and context of treatment, including instructions to practitioners, and information and explanations to patients | No |
| **5. Practitioner background** | 5) Description of participating acupuncturists (qualification or professional affiliation, years in acupuncture practice, other relevant experience) | Traditional Medicine doctors who have had TEA treatment experience of 10 years |
| **6. Control or comparator interventions** | 6a) Rationale for the control or comparator in the context of the research question, with sources that justify this choice | Sham Thread-embedding acupuncture (STEA): All procedure of STEA group, including acupoints and size of TEA will be same as that of TEA group. However, thread-removed TEA will be used for STEA group instead of normal TEA, and removing procedure of thread will be performed aseptic and secretly for patient-blinding and prevention of infection |
|  | 6b) Precise description of the control or comparator. If sham acupuncture or any other type of acupuncture-like control is used, provide details as for Items 1 to 3 above. |  |
