# Supplementary material for: Effect on Satisfactory Seizure Control and Heart Rate Variability of Thread-Embedding Acupuncture for Drug-Resistant Epilepsy: A Patient-Assessor Blinded, Randomized Controlled Trial
Source: Behav Neurol. 2023 Sep 19;2023:5871991. doi: 10.1155/2023/5871991 (PMC10522444; doi:10.1155/2023/5871991)
Supplement: Supplementary Materials — Table S1: details of acupuncture treatment based on the STRICTA 2010 checklist. Table S2: the CONSORT 2010 checklist with the Nonpharmacological Trials Extension to CONSORT. [file 5871991.f1.zip › update_Table S2. The CONSORT 2010 checklist.docx]

**Table S2. The CONSORT 2010 checklist with the Non-pharmacological Trials Extension to CONSORT**

| **Section/Topic** | **Item #** | **CONSORT 2010 Statement*: Checklist item[10]. Describe:** | **Additional items from the Non-pharmacological Trials Extension to CONSORT. Add:** | **Reported on Page No** |
| --- | --- | --- | --- | --- |
| ***TITLE AND ABSTRACT*** | | | | |
|  | 1.a | Identification as a randomized trial in the title | In the abstract, description of the experimental treatment, comparator, care providers, centres and blinding status. | 1-3 |
|  | 1.b | Structured summary of trial design, methods, results, and conclusions; for specific guidance see CONSORT for Abstracts |  | 24-37 |
| ***INTRODUCTION*** | | | | |
| Background and objectives | 2.a | Scientific background and explanation of rationale |  | 41-72 |
|  | 2.b | Specific objectives or hypotheses |  | 73-76 |
| **METHODS** | | | | |
| Trial design | 3.a | Description of trial design (e.g., parallel, factorial) including allocation ratio |  | 79-89 |
|  | 3.b | Important changes to methods after trial commencement (e.g. eligibility criteria), with reasons |  | No |
| Participants | 4.a | Eligibility criteria for participants | When applicable, eligibility criteria for centers and those performing the interventions. | 90-102 |
|  | 4.b | Settings and locations where the data were collected |  | 91-92 |
| Interventions | **5** | **The interventions for each group with sufficient details to allow replication, including how and when they were actually administered** | **Precise details of both the experimental treatment and comparator - see Table S1 for details** | **See Table S1** |
| Outcomes | 6.a | Completely defined pre-specified primary and secondary outcome measures, including how and when they were assessed |  | 147-163 |
|  | 6.b | Any changes to trial outcomes after the trial commenced with reasons |  | No |
| Sample size | 7.a | How sample size was determined | When applicable, details of whether and how the clustering by care providers or centers was addressed. | 139-142 |
|  | 7.b | When applicable, explanation of any interim analyses and stopping guidelines |  | 164-169 |
|  | | | | |
| *Sequence generation* | 8.a | Method used to generate the random allocation sequence | When applicable, how care providers were allocated to each trial group. | 103-114 |
|  | 8.b | Type of randomization; details of any restriction (e.g., blocking and block size) |  | 103-114 |
| *Allocation concealment* | 9 | Mechanism used to implement the random allocation sequence (e.g., sequentially numbered containers), describing any steps taken to conceal the sequence until interventions were assigned |  | 103-114 |
| *Implementation* | 10 | Who generated the random allocation sequence, who enrolled participants, and who assigned participants to interventions |  | 103-114 |
| Blinding | 11.a | If done, who was blinded after assignment to interventions (e.g. participants, care providers, those assessing outcomes) and how | Whether or not those administering co-interventions were blinded to group assignment. If blinded, method of blinding and description of the similarity of interventions. | 103-114 |
|  | 11.b | If relevant, description of the similarity of interventions |  | 103-114 |
| Statistical methods | 12.a | Statistical methods used to compare groups for primary and secondary outcomes | When applicable, details of whether and how the clustering by care providers or centers was addressed. | 170-178 |
|  | 12.b | Methods for additional analyses, such as subgroup analyses and adjusted analyses |  | No |
| **RESULTS** | | | | |
| Participant flow (A diagram is strongly recommended) | 13.a | For each group, the numbers of participants who were randomly assigned, received intended treatment, and were analyzed for the primary outcome | The number of care providers or centers performing the intervention in each group and the number of patients treated by each care provider or in each center. | 181-190 |
|  | 13.b | For each group, losses and exclusions after randomization, together with reasons |  | 181-190 Figure 2 |
| Implementation of intervention |  |  | Details of the experimental treatment and comparator as they were implemented. | 181-190 Figure 2 |
| Recruitment | 14.a | Dates defining the periods of recruitment and follow-up |  | 181-190 Figure 2 |
|  | 14.b | Why the trial ended or was stopped |  | 181-190 Figure 2 |
| Baseline data | 15 | A table showing baseline demographic and clinical characteristics for each group | When applicable, a description of care providers (case volume, qualification, expertise, etc.) and centers (volume) in each group. | 186-190  Table 1 |
| Numbers analyzed | 16 | For each group, number of participants (denominator) included in each analysis and whether the analysis was by original assigned groups |  | 181-190 Figure 2 |
| Outcomes and estimation | 17.a | For each primary and secondary outcome, results for each group, and the estimated effect size and its precision (e.g., 95% confidence interval) |  | 191-213 |
|  | 17.b | For binary outcomes, presentation of both absolute and relative effect sizes is recommended |  | 191-213 |
| Ancillary analyses | 18 | Results of any other analyses performed, including subgroup analyses and adjusted analyses, distinguishing pre-specified from exploratory |  | No |
| Harms | 19 | All important harms or unintended effects in each group; for specific guidance see CONSORT for Harms [60] |  | *Safety assessment*  209-213 |
| **DISCUSSION** | | | | |
| Limitations | 20 | Trial limitations, addressing sources of potential bias, imprecision, and, if relevant, multiplicity of analyses |  | 269-279 |
| Generalizability | 21 | Generalizability (external validity, applicability) of the trial findings | Generalizability (external validity) of the trial findings according to the intervention, comparators, patients and care providers and centers involved in the trial. | 216-220 |
| Interpretation | 22 | Interpretation consistent with results, balancing benefits and harms, and considering other relevant evidence | In addition, take into account the choice of the comparator, lack of or partial blinding, unequal expertise of care providers or centers in each group. | 221-268 |
| ***Other Information*** | | | | |
| Registration | 23 | Registration number and name of trial registry |  | 82-86 |
| Protocol | 24 | Where the full trial protocol can be accessed, if available |  | - |
| Funding | 25 | Sources of funding and other support (e.g., supply of drugs); role of funders |  | 299 |

***Citation:*** *MacPherson H, Altman DG, Hammerschlag R, Youping L, Taixiang W, White A, Moher D; STRICTA Revision Group. Revised STandards for Reporting Interventions in Clinical Trials of Acupuncture (STRICTA): extending the CONSORT statement. PLoS Med. 2010 Jun 8;7(6):e1000261*
